# R-STDP Spiking Neural Network Architecture for Motion Control on a Changing Friction Joint Robotic Arm

**DOI:** 10.3389/fnbot.2022.904017

**Published:** 2022-05-18

**Authors:** Alejandro Juarez-Lora, Victor H. Ponce-Ponce, Humberto Sossa, Elsa Rubio-Espino

**Affiliations:** Instituto Politécnico Nacional, Centro de Investigación en Computación, Mexico City, México

**Keywords:** neuromorphic, robotics, reinforcement learning, STDP, reward modulation, control theory, applications

## Abstract

Neuromorphic computing is a recent class of brain-inspired high-performance computer platforms and algorithms involving biologically-inspired models adopting hardware implementation in integrated circuits. The neuromorphic computing applications have provoked the rise of highly connected neurons and synapses in analog circuit systems that can be used to solve today's challenging machine learning problems. In conjunction with biologically plausible learning rules, such as the Hebbian learning and memristive devices, biologically-inspired spiking neural networks are considered the next-generation neuromorphic hardware construction blocks that will enable the deployment of new analog *in situ* learning capable and energetic efficient brain-like devices. These features are envisioned for modern mobile robotic implementations, currently challenging to overcome the pervasive von Neumann computer architecture. This study proposes a new neural architecture using the spike-time-dependent plasticity learning method and step-forward encoding algorithm for a self tuning neural control of motion in a joint robotic arm subjected to dynamic modifications. Simulations were conducted to demonstrate the proposed neural architecture's feasibility as the network successfully compensates for changing dynamics at each simulation run.

## 1. Introduction

Spiking neural networks (SNNs), also called the third generation of neural networks, represent a new design paradigm where some biological neural dynamics are replicated, with similar energy efficiency and *in situ* learning capabilities, as seen in living organisms, whereas hardware miniaturization is feasible. Neuromorphic computing (Saxena et al., 2018; Kendall and Kumar, 2020) emerges as an effort to create built-in neural hardware, emulating the neuronal impulsive-like electrical activity and *in-situ* synaptic learning in analog devices. Therefore, neuron dynamics have to be translated into circuit proposals to achieve these behaviors. As for the synapses, where learning occurs in biological brains, memristors are taking their role as the electrical element counterpart (Zhang et al., 2021). These devices, theorized by Leon Chua, relate flux with charge, resulting in a variable resistor. The conductivity is given by how much current has flowed between its ports in a determined period. Therefore, its conductance serves as the synaptic weight which can be tuned by applying current (Yue and Parker, 2019). At Zamarreño-Ramos et al. (2011), an exploration into how neurons and memristors can be interconnected as an array scheme to achieve large scale spiking systems, using synaptic time-dependant plasticity (STDP), is presented. Since then, several proposals have been presented. Recently, a memristor analog crossbar circuit is used to emulate a single layer perceptron for the MNIST image classification problem (Kim et al., 2021). In Shi et al. (2021), a circuit proposed to manage reward modulation is presented, setting the building blocks for implementation.

Cutting-edge neuromorphic implementations still demand going deeper into studying the neuron dynamics and plausible learning methods since the non-differentiable nature of the neuron dynamic doesn't allow the use of the well-known backpropagation synaptic weight adjustment; widely employed in ordinary artificial neural networks (ANN). Therefore, there are some open challenges to address before constructing high-performance neuromorphic devices, as well as encoding and decoding information techniques. According to Hu et al. (2022), learning algorithms used in SNNs are summarized in:

- Modified gradient-descendent-based algorithms: As neuron models are non-differentiable, some modifications are pertinent to achieve the classical backpropagation learning rule, employed in most of the ANNs, i.e., SpikeProp (Kheradpisheh and Masquelier, 2020).
- Algorithms using a spike train kernel: Where an error function is used to compute and update synaptic weights, using a spike train kernel, i.e., SPAN (Mohemmed et al., 2012).
- Algorithms using synaptic plasticity: Based on Hebbian learning, the synaptic weights tuning is given by the correlation of pre and post-synaptic spikes. In STDP, the modification of the neural strength connections is performed as the learning process occurs, as in biological brains (Hao et al., 2020).

Synaptic plasticity phenomena explain how learning is conducted in biological brains, enhancing conductivity between *neurons that fire together, wire together*, and deprecating those unused connections.

On other hand, information, usually shaped as an analog signal, has to be encoded into the neuron spike domain. The scientific community is still debating how information from the environment is converted into electrical neural activity. According to Dupeyroux et al. (2021), neuron spike coding methods can be classified into three categories.

- *Population encoding*: A group of *n* neurons, each one with different characterization (i.e., different τ_*m*_, *R*_*m*_, *C*_*m*_), is set to be excited about an input current. As a result, at a given time-step, some neurons will spike faster than others. The characterization of neurons is made in such a way the domain of the input signal is distributed between the *n* neurons, using *tuning curves* (Voelker and Eliasmith, 2020).
- *Rate-based encoding*: One neuron is used to encode the variation of the input signal ∈ [*I*_*min*_, *I*_*max*_]. As larger an input signal gets, the spiking frequency of the neuron increases. A minimal input current traduces into a minimum spike frequency, inside a frequency interval ∈ [*f*_*min*_, *f*_*max*_].
- *Temporal encoding*: Also called pulse coding, produces spikes according to a temporal change of the input signal. This is, if an input signal is constant, no spikes are produced, even if the signal is large. As soon the signal increases or decreases, spikes will be emitted.

While population encoding reaches the best performance, its efficiency is reduced, as it needs a huge amount of resources (neurons) in order to be implemented. Rate-based encoding has become the standard, but it presents the need to spike even with a zero input signal, increasing the power consumption. Besides, it can't encode negative values, as seen in Bing et al. (2019a), where a negative input signal has to be fed as its absolute value. The temporal encoding provides a time-based method, providing more information capacity per synaptic event, and it is supported by neurophysiological studies in auditory and visual processing in the brain (Guo et al., 2021). SNNs can send data encoded as the timing of spikes occurrences, allowing fast and low energy consumption hardware implementation, applicable to real-world robotics problems. Furthermore, SNNs are more prominent than non-spiking ANN as they profit from temporal stimulus information, referring to the precise timing of events that allows obtaining and processing of information.

Spiking neural networks on robotic design systems are a promising research topic as online learning, and huge computational capacities are commonly required in this field. For instance, at Chen et al. (2020), an SNN controls a 4-DOF (Degree of Freedom) manipulator arm using population encoding and a proposed learning rule. Bing et al. (2019b) use a reward-modulation learning rule to teach a differential robot how to track a path, using rate-based encoding to do conversion of visual input into spiking activity. A similar task is studied numerically by Bing et al. (2019b), controlling a snake's movement instead. Lu et al. (2021) achieve obstacle avoidance for an Ackerman-type mobile robot, using two neurons and two synapses, implemented on a digital development board. Bing et al. (2019a) achieve obstacle avoidance and goal-reaching for a differential robot, implementing separate neural control structures for each task. Over these articles, while control is achieved based on interaction with the environment, changing dynamics in the robot produced by weathering in the joints or unknown environmental perturbances are not addressed. A typical control strategy for a robotic open-chain manipulator requires re-tuning each time friction or mass on the robot changes, affecting performance. This article proposes an SNN architecture that learns how to reconstruct an input signal. Inspired by control theory, this structure is then used in a control loop, using the same input signals in a PID, but fed into the structure in order for the synaptic weights to evolve over changing dynamics on a 1-DOF robotic arm.

The document is structured as follows: Section 2 describes the control problem to be tackled, neuron and synapse dynamics, and how these are ensembled for a controlling proposal. Section 3 shows the simulation results of the proposed SNN implemented in 1-link. Section 4 discusses results, advantages, and drawbacks, while at last, Section 5 is devoted to conclusion and future study.

## 2. Materials and Methods

### 2.1. Control Problematic

According to Craig (1986); Lynch (2017), the dynamics of an open chain robotic manipulator can be written in joint space as:


(1) $$\begin{array}{l} \tau =M\left(q\right)\ddot{q}+C\left(q,\dot{q}\right)\dot{q}+g\left(q\right) \end{array}$$

Where $q={\left[\theta_{1},\theta_{2},...,\theta_{n}\right]}^{T}$ are the joint angles of the robotic arm with *n* DOFs, *M*(·) stands for inertia matrix terms, *C*(·) is the Coriolis's matrix and friction dynamics, *g*(·) are gravity compensation terms and τ = [τ^1^, τ^2^, ..., τ^*n*^]^*T*^ means the torque control for each joint. Typically, PID control strategies are the standard. Based on the desired state *x*_*d*_(*t*), a tracking error *q*_*e*_ = *q*_*d*_ − *q* is defined, setting the control input τ(*t*) as:


(2) $$\begin{array}{l} \tau \left(t\right)=K_{P}\theta_{e}+K_{i}\int \theta_{e}\left(t\right)dt+K_{d}{\dot{\theta }}_{e} \end{array}$$

At Equation (2), $K_{P}\in R^{n\times n},K_{d}\in R^{n\times n},K_{i}\in R^{n\times n}$ are the gain matrix for proportional, derivative, and integral control, which elements are zeros except in the diagonal. This strategy is the function of the tracking error, which on zero, there will be no control output. Consider:


(3) $$\begin{array}{l} \tau =\tilde{M}\left(q\right)\ddot{q}+\tilde{C}\left(q,\dot{q}\right)+\tilde{g}\left(q\right) \end{array}$$

Here, $\tilde{M},\tilde{C},\tilde{g}$ represents our model representation of the plant, and it is perfect if $\tilde{M}\left(\ddot{q}\right)=M\left(\ddot{q}\right)$, $\tilde{C}\left(q,\dot{q}\right)=C\left(q,\dot{q}\right)$, and $\tilde{g}\left(q\right)=g\left(q\right)$. Therefore, if the control loop works on the estimation, it would work for the real model. Usually, in the development process of a robot controller, *K*_*p*_, *K*_*i*_, and *K*_*d*_ are tuned for initial $\tilde{M},\tilde{C}$, and $\tilde{g}$. This becomes a problem as the robot's weathering modifies its dynamic properties, such as friction. Or perhaps, mass changes over time, as seen in biological limbs in living creatures.

### 2.2. Spiking Neural Network Modeling

In order to describe the proposed structure, a review of how a neuron generates spikes, how synapses store learning, and how to generate reward signals is presented.

#### 2.2.1. Neuron Modeling

As an input stimulus is provided to the neuron cell, shaped as an input current, the membrane's potential *v*_*m*_ increases. Once it overpasses a threshold voltage *v*_*th*_, the neuron produces a spike, and then it immediately resets its membrane potential to a reset voltage *v*_*reset*_. The neuron cannot fire again until a certain refractory period has elapsed. Some differential equation models illustrate these neural dynamics with high biological plausibility but prohibitive computational cost such as Hodgin and Huxley or Izhikevich models (Izhikevich, 2004; Valadez-Godínez et al., 2020). Nonetheless, others with a lesser plausibility can compute the membrane potential with less effort degrading the accuracy, but are still useful as a good model approximation, due that spikes generation with the same characteristics as biological neurons might not be necessary for circuit implementations, such as the *Leaky Integrate and Fire (LIF)* (Lu et al., 2021) model, given by:


(4) $$\begin{array}{l} \tau_{m}\frac{dv_{m}\left(t\right)}{dt}=-v_{m}\left(t\right)+E_{l}+R_{m}I_{syn} \end{array}$$

At Equation (4), *v*_*m*_(*t*) represents the neuron's membrane potential, *E*_*l*_ is the resting potential of the neuron, *R*_*m*_ membrane resistance, τ_*m*_ = *R*_*m*_*C*_*m*_ is the decay time for *v*_*m*_(*t*), being *C*_*m*_ the neuron's membrane capacitance. *I*_*syn*_ stands for the injected current to the neuron. Each time a spike arrives at the neuron, *I*_*syn*_ increases. On the other hand, if no spikes arrive at the neuron, the current decays. This phenomenon is described by LIF conductance-based model (Hao et al., 2020; Lu et al., 2021), composed of Equations (4 and 5):


(5) $$\begin{array}{l} \tau_{m}\frac{dI_{syn}}{dt}=-I_{syn}+C_{m}\overset{N}{\underset{i}{\sum }}w_{ij}\delta \left(t-t_{i}^{f}\right) \end{array}$$

**Figure 1:**
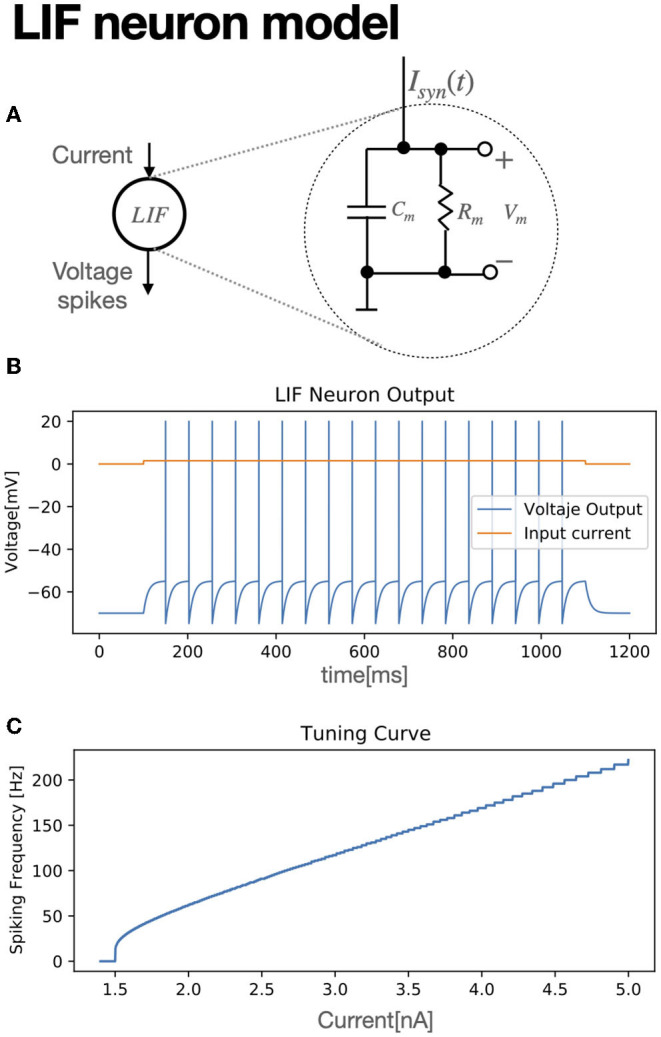
LIF model **(A)** diagram of a spiking LIF neuron. **(B)** Spiking activity for a LIF neuron with *I*_*syn*_ = 1.51 *nA*. **(C)** Tuning curve of the neuron model. Neuron parameters are in Table 1.

In Equation (5), *w*_*ij*_ is the synapse strength value between a presynaptic, *i* − *th*, neuron and a postsynaptic, *j* − *th*, neuron. As for each postsynaptic neuron, there can be *N* presynaptic neurons connected, $t_{i}^{f}$ is then a vector with firing times from each of the *N* presynaptic neurons. δ is the Kronedecker delta function, which δ(*x*) = 1 for *x* = 0 and δ(*x*) = 0 for *x*≠0. Equation (5) assumes all presynaptic spikes have been produced at time *t*. For each time a new spike happens, $t_{i}^{f}=t$, therefore, $\delta \left(t-t_{i}^{f}\right)=1$. Once the neuron threshold voltage *v*_*th*_ is over-passed, the neuron spikes, emitting a pulse of magnitude *v*_*spike*_, then, the neuron resets to a reset potential *v* = *v*_*reset*_ and it starts integrating again. Figure 1A shows the LIF structure model, while its spiking activity for a given fixed and variable input current is shown at Figures 1B,C, respectively.

**Table 1:** Neuron and synapse modeling parameters.

**Model**	**Parameters**	**Value**
LIF neuron	Membrane resistance	*R*_*m*_ = 10 *M*Ω
	Membrane's capacitance	*C*_*m*_ = 1 *nF*
	Time decay membrane	τ_*m*_ = 0.010 *s*
	Resting voltage	*E*_*l*_ = −70 *mV*
	Reset voltage	*V*_*reset*_ = −75 *mV*
	Spike voltage	*V*_*spike*_ = 20 *mV*
	Threshold voltage	*V*_*th*_ = −55 *mV*
RSTDP synapse	LTP scaling	*A*_+_ = 1
	LTD scaling	*A*_−_ = −1
	Elegibity trace scale	τ_*E*_ = 0.010 *s*
	Min. Synaptic weight	*w*_*min*_ = 1
	Max. Synaptic weight	*w*_*max*_ = 1,000

#### 2.2.2. Synaptic Modeling

Once we define how neurons produce spikes, we will expose how synaptic strength is adjusted. STDP (Bing et al., 2019b) is an unsupervised learning algorithm (based on the Hebbian learning rule) for SNN. It describes how synaptic weights are strengthened or weakened according to neural spike activity, and it has demonstrated plausibility over conducted experiments in biological systems. First, a synaptic weight value is randomly assigned for each defined synapse. Then, the time difference between pre and post-synaptic firing times Δ*t* = *t*_*post*_ − *t*_*pre*_ is computed and it determines the rate of change Δ*w* on the synaptic weight *w* as:


(6) $$\begin{array}{l} STDP\left(\Delta t\right)=\{\begin{array}{cc} A_{+}e^{-\Delta t/\tau_{post}} & \;\Delta t\geq 0\\ A_{-}e^{-\Delta t/\tau_{pre}} & \;\Delta t<0 \end{array} \end{array}$$

(7) $$\begin{array}{l} ẇ=\underset{t_{pre}}{\sum }\underset{t_{post}}{\sum }STDP\left(\Delta t\right) \end{array}$$

Here, *A*_+_, *A*_−_ are scaling constants depicting whether our synaptic weight has been incremented (*Long Term Potentiation* LTP) or decremented (*Long Term Depression* LTD). τ_*pre*_, τ_*post*_ are positive and negative constants representing decay time. Once again, these equations imply all spikes have been produced. Since *neurons do not have a memory of all their fired spikes* (Bing et al., 2019a), Equation (6) can be rewritten in the function of the last firing time (Morrison et al., 2008; Gerstner et al., 2018). This results in the following expressions:


(8) $$\begin{array}{l} STDP\left(t-t_{pre/post}\right)=\{\begin{array}{c} A_{+}\delta \left(t-t_{pre}\right)\\ A_{-}\delta \left(t-t_{post}\right) \end{array} \end{array}$$

As each time a presynaptic spike *t*_*pre*_ is produced, *t*_*pre*_ = *t* and δ(*t* − *t*_*pre*_) = 1. With each postsynaptic spike, *t*_*post*_ = *t* and δ(*t* − *t*_*post*_) = 1. Next, we define an eligibility trace *E*_*jk*_ for each synapse between a presynaptic neuron *j* and a post-synaptic neuron *k* as:


(9) $$\begin{array}{l} {Ė}_{jk}\left(t\right)=-\frac{E_{jk}}{\tau_{E}}+STDP\left(t-t_{pre/post}\right) \end{array}$$

This expression computes synaptic weight changing history, generated by the collected spikes. To control the sensitivity of the plasticity to delayed reward, an exponential τ_*E*_ = τ_*pre*_ = τ_*post*_ constant for *E*_*jk*_(*t*) is defined. This implies a symmetric learning rate for LTD and LTP (Taherkhani et al., 2020). Change in synaptic weights is obtained by integrating (Equation 9). The *reward modulated STDP*, or R-STDP learning rule model, integrates the reinforcement learning paradigm in SNNs, modifying the STDP algorithm based on dopamine effects for learning in biological brains (Framaux and Gerstner, 2016). Consider:


(10) $$\begin{array}{l} {ẇ}_{jk}\left(t\right)=R_{jk}\left(t\right)\times E_{jk}\left(t\right) \end{array}$$

Here, $R_{jk}\left(t\right)\in R^{n\times 1}$ is a reward signal for the synapses between layer a *j* − *th* and *k* − *th* layer in a network, bounded inside [−1, 1], which enables or disables synaptic modification (called learning), and it is defined by interaction with the environment as a function of an objective (i.e., the desired path, desired position, desired action). It is worth mentioning that, when *R*_*jk*_ = 1, the R-STDP rule equals STDP, as Equation (7) equals Equation (10). When *R*_*jk*_ is equal to 0, learning is inhibited.

#### 2.2.3. Encoding and Decoding Between Continuous and Spike Domains

Step forward encoding (SF henceforth), described in Kasabov et al. (2016) and Dupeyroux et al. (2021), is considered a temporal encoding algorithm, as it converts the variation of an input signal to spikes. The module for the step forward encoding contains two outputs ports *Out*_+_, *Out*_−_, and an input port, *s*_*in*_, which is compared with a baseline value *s*_*b*_. If the incoming signal is bigger than a certain predefined threshold value *s*_*th*_ (this is: *s*_*in*_ > *s*_*b*_ + *s*_*th*_), then a spike will be produced over *Out*_+_. On the contrary, if the signal has decreased (*s*_*in*_ < *s*_*b*_ − *s*_*th*_), a spike will be produced in *Out*_−_. As the spike's domain is always positive, the emitted spikes can be processed by SNNs representing positive and negative changes in value. The procedure herein is shown in Algorithm 1.

**Algorithm 1:**
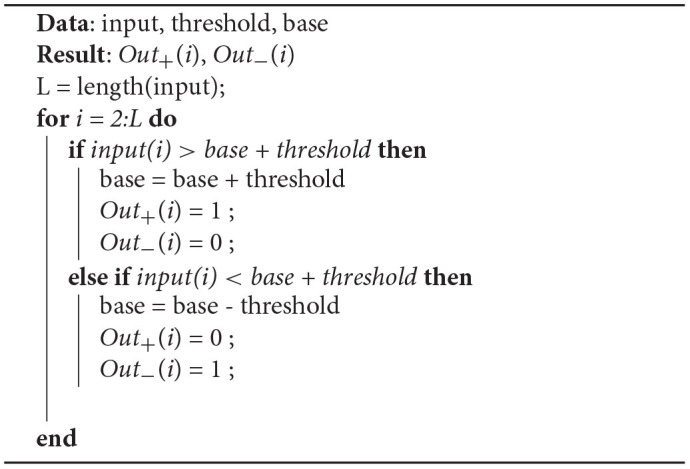
Modified step forward encoding algorithm.

**Figure 2:**
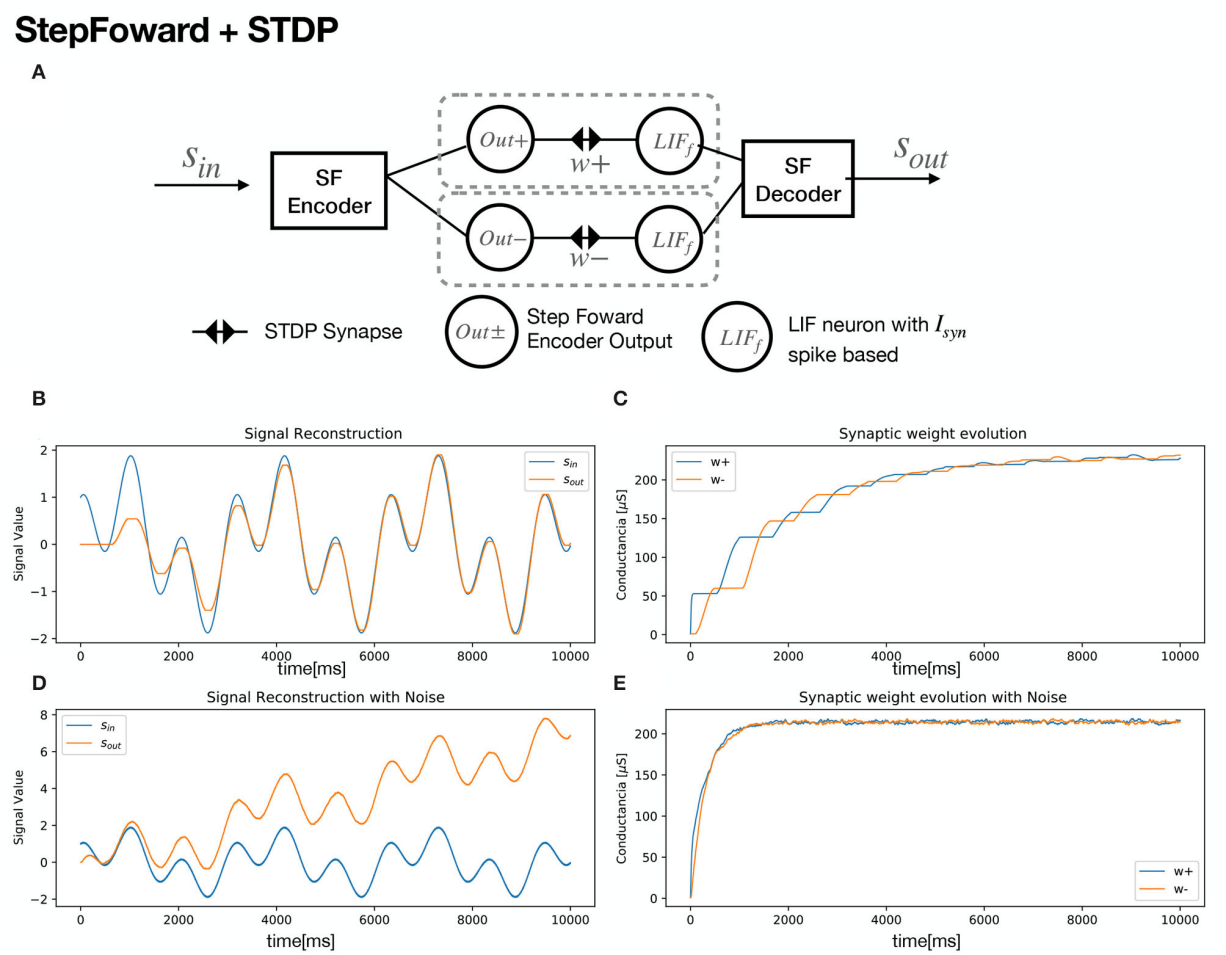
Step Forward + STDP Neural structure. **(A)** Schematic of the structure. **(B)** Sinusoidal Signal *s*_*in*_ = sin(ω*t*) + cos(3ω*t*) being reconstructed. **(C)** Synaptic Weight evolution. **(D)** Gaussian noise with σ = 0.01 was added to foster synaptic update. **(E)** Synaptic Weight evolution with added noise. Simulation Parameters are available at Table 2.

**Figure 3:**
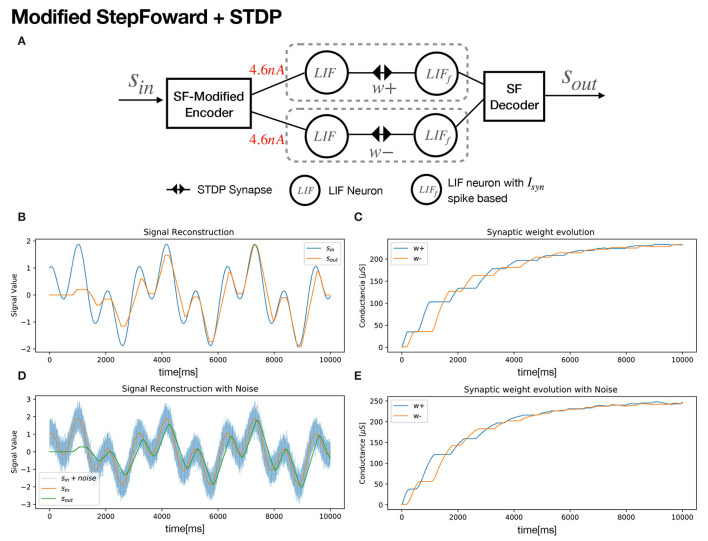
Modified Step Forward + STDP Neural structure. **(A)** Schematic of the structure. **(B)** Sinusoidal Signal *s*_*in*_ = sin(ω*t*) + cos(3ω*t*) being reconstructed. **(C)** Synaptic Weight evolution. **(D)** Gaussian noise with σ = 0.3 was added to foster synaptic update. **(E)** Synaptic Weight evolution with added noise. Simulation parameters are available at Table 3.

A neural structure proposal for exploiting SF encoding with SNN and STDP is shown in Figure 2A. An input signal is fed to an encoder and the decoded output signal tends to match the original, as the synaptic weights get updated (Figure 2B). For signal growth, learning in the *w*+ synapse occurs. Once the signal decreases, an update for negative synapse *w*− begins (Figure 2C). In order to foster a quick synaptic weight adjustment in both synapses, Gaussian noise was added to the input signal *s*_*in*_ (Figure 2D), with a SD of σ = 0.01. As a secondary effect, accumulation in the decoder's output signal takes place, as seen in Figure 2E. In order to harness the low-pass filter dynamics of the LIF neuron model, a slight modification is proposed. Instead of spikes, a given current *I*_*c*_ is sent as the encoder outputs. Figure 3 shows the same signal reconstruction obtained with the proposed modifications, setting *I*_*c*_ = 4.6*nA* as current input to a LIF neuron which, according to its tuning curve (refer to Figure 1C), it would produce spikes at a frequency of 200 Hz. Signal reconstruction is achieved. Moreover, Gaussian noise with σ = 0.3 is added to the input signal, achieving signal reconstruction and filtering, as seen in Figure 3D.

**Table 2:** Step Foward + STDP Encoding simulation.

**Model**	**Parameters**	**Value**
Simulation parameters	Time step	*dt* = 0.1*ms*
	Signal angular velocity	ω = 2*rad*/*s*
	Total time simulation	10*s*
SF encoder	Threshold	*s*_*th*_ = 0.02
	Initial base value	*s*_*b*_ = 0
SF decoder	Threshold	*s*_*th*_ = 0.02
	Initial base value	*s*_*b*_ = 0

**Table 3:** Modified Step Foward + STDP Encoding simulation.

**Model**	**Parameters**	**Value**
Simulation parameters	Time step	*dt* = 0.1 *ms*
	Signal angular velocity	ω = 2 *rad*/*s*
	Added gaussian noise	10%
	Total time simulation	10 *s*
SF encoder	Threshold	*s*_*th*_ = 0.0005
	Initial base value	*s*_*b*_ = 0
	Current output	*I*_*c*_ = 4.6 *nA*
SF decoder	Threshold	*s*_*th*_ = 0.025
	Initial base value	*s*_*b*_ = 0

### 2.3. Self Tuning SNN Controller Proposal

**Figure 4:**
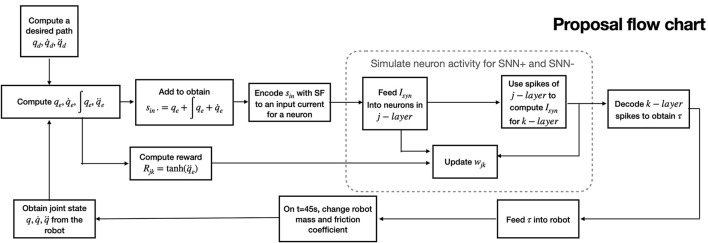
SNN-PID control proposal flow chart showing steps for implementation.

**Figure 5:**
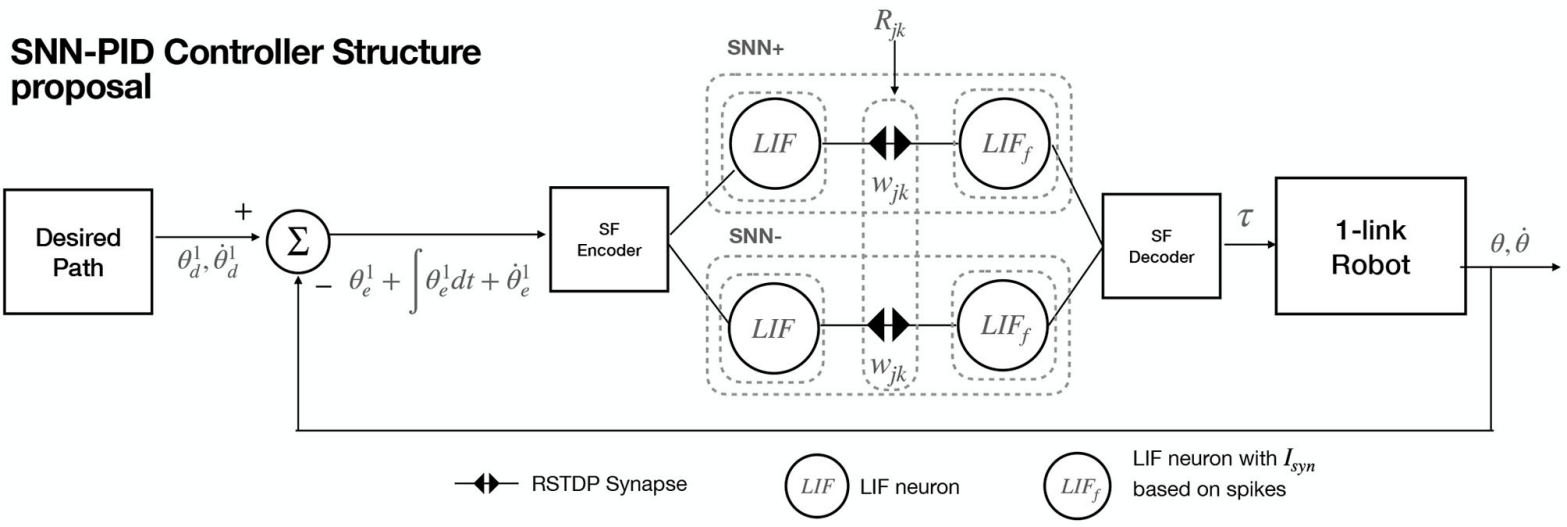
SNN-PID controller neural structure for 1-DOF robot pendulum.

The objective is to create an SNN structure that enables the learning of the robot's dynamics and reconstructs the necessary torque control output based on Equation (2). In order to take advantage of synaptic plasticity properties, tuning PID control parameters *K* on the fly is performed. The procedure steps are shown in Figure 4 and described in detail upnext. First, a module computes the desired path, using a cubic polynomial trajectory planning generation Algorithm (Craig, 1986; Spong et al., 2005), with initial and final points randomly defined between the joint's boundaries, and initial and final desired velocities set to zero. Next, $q_{e},\int q_{e}dt,{\dot{q}}_{e}$ are computed and added; then, they are applied to an SF encoder module, which out is sent to an SNN processing positive changes (called SNN+), and another for negative changes (called SNN-). Both networks share the same structure, as SNN+ and SNN- are intended to process the necessary signal increments and decrements, respectively. These networks are composed of two layers *j* − *th* and *k* − *th* composed of *n* LIF neurons each (same amount of DOFs in the robot), modeled by Equations (4), (5). Between *j* − *th* and *k* − *th* layers, there are $w_{jk}\in R^{n\times n}$ synapse matrices with randomly initialized weight values between minimum and maximum synaptic values [*w*_*min*_, *w*_*max*_]. For all synapses, its value will be modified accordingly to Equations (8–10). Each output spike from the neurons of the *k* − *th* layer in SNN+ and SNN- serves as input for the *n* SF decoders, which output corresponds to each torque input signal for the robot. The proposed structure is shown in Figure 5.

## 3. Results

### 3.1. 1-DOF SNN Simulation Implementation

For a 1-link robot (a pendulum), its non-linear model has a shape like in Equation (1) and is given by:


(11) $$\begin{array}{l} \tau =ml^{2}\ddot{\theta }+mgl\sin\left(\theta \right)+ml^{2}k\dot{\theta } \end{array}$$

Where *m* stands for the arm's weight, *l* its length, *g* is the gravity acceleration term, *k* is the viscous friction in the joint, θ ∈ [θ_*max*_, θ_*min*_] is the joint angle, τ represents torque in the robot's joint, acting as the input control signal to the system.

**Figure 6:**
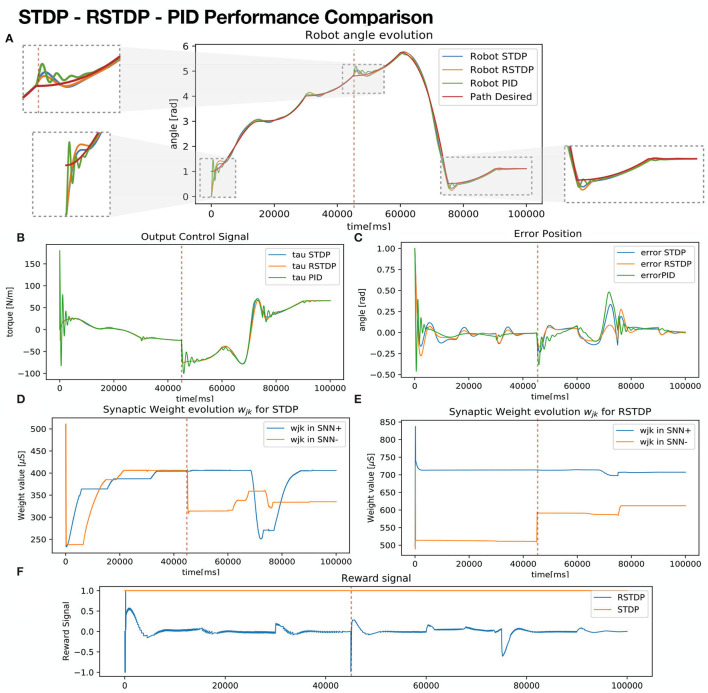
1-Link robot controller performance comparison. **(A)** Robot Angle evolution for each robot, SNN-RSTDP: with $R_{jk}=\tanh{\ddot{q}}_{e}$ (Yellow), SNN-STDP: proposal with *R*_*jk*_ = 1 (Blue), PID-Controller tuned with Ziegler Nichols (Green), compared against reference (Red). **(B)** Control output of each controller. **(C)** Error position: difference against the reference. **(D)** Synaptic weights evolution for constant reward robot (SNN-STDP). **(E)** Synaptic weight evolution for the rewarded robot (SNN-RSTDP). **(F)** Reward signal for both SNN proposals.

Figure 6 shows simulation results comparing performance between three controllers:

- SNN-STDP: proposed model with fixed reward signal *R*_*jk*_ = 1.
- SNN-RSTDP: proposed model with reward signal *R*_*jk*_ given as a function of acceleration error ${\ddot{q}}_{e}$.
- A manually tuned PID controller, tuned by the Ziegler Nichols technique (Ogata, 2010)

For the SNN-RSTDP controller, a bounded reward signal delimited by [−1, 1], is given next:


(12) $$\begin{array}{l} R_{jk}=\text{tanh}\left({\ddot{q}}_{e}\right) \end{array}$$

For each episode of length 15*s*, the desired path is computed, selecting initial and final positions randomly, but setting the final position of the current episode as the initial position for the next episode. The proposed SNN quickly tunes itself. At *t* = 45*s* (refer to red dotted vertical line), the link's joint friction coefficient *k* and weight *m* increase to a new *k*_*new*_ and *m*_*new*_ values. From *t* = 90*s* to *t* = 100*s*, $q_{d}=constant,{\dot{q}}_{d}=0$.

In Figures 6A,B, it can be seen that robot angle runs evolve smoothly on both RSTDP and STDP controlled robots, in contradistinction from PID controlled robots, in which evolution oscillates more. Besides, in Figure 6C, it can be seen at the output control for the PID presents jittering, which in real scenarios would produce fatigue on the motor, decreasing its lifespan. In order to analyze the variance of the tracking task, a *root mean squared error (RMSE)* metric signal (Petro et al., 2020), which is intended to be minimized, is defined as:


(13) $$\begin{array}{l} RMSE_{q}=\sqrt{\frac{\sum_{t=1}^{N}{\left(q-q_{d}\right)}^{2}}{N}} \end{array}$$

Where *N* is the number of all timesteps along with the experiment. Similar values $RMSE_{\dot{q}}$, $RMSE_{\ddot{q}}$ can be obtained using velocities $\dot{q}$ and accelerations $\ddot{q}$ instead. Table 4 shows the mean *RMSE* values for a hundred iterations with random desired trajectories, comparing the position, velocity, and acceleration for each of the three used controllers. It can be seen that RSTDP and STDP controllers achieve better performance, having lower RMSE values. $RMSE_{\ddot{q}}$ presents the worst metrics for the PID controller, explaining the jittering for the torque output, which is the function of the acceleration of the robot.

**Table 4:** RMSE analysis result comparison for STDP-SNN, RSTDP-SNN, and PID controllers.

**Signal**	**RSTDP**	**STDP**	**PID**
Position	**0.101231**	0.12089	0.116812
Velocity	**0.116812**	0.21960	0.33481
Acceleration	0.43922	**0.400311**	1.49198

*Best cases are in bold*.

**Figure 7:**
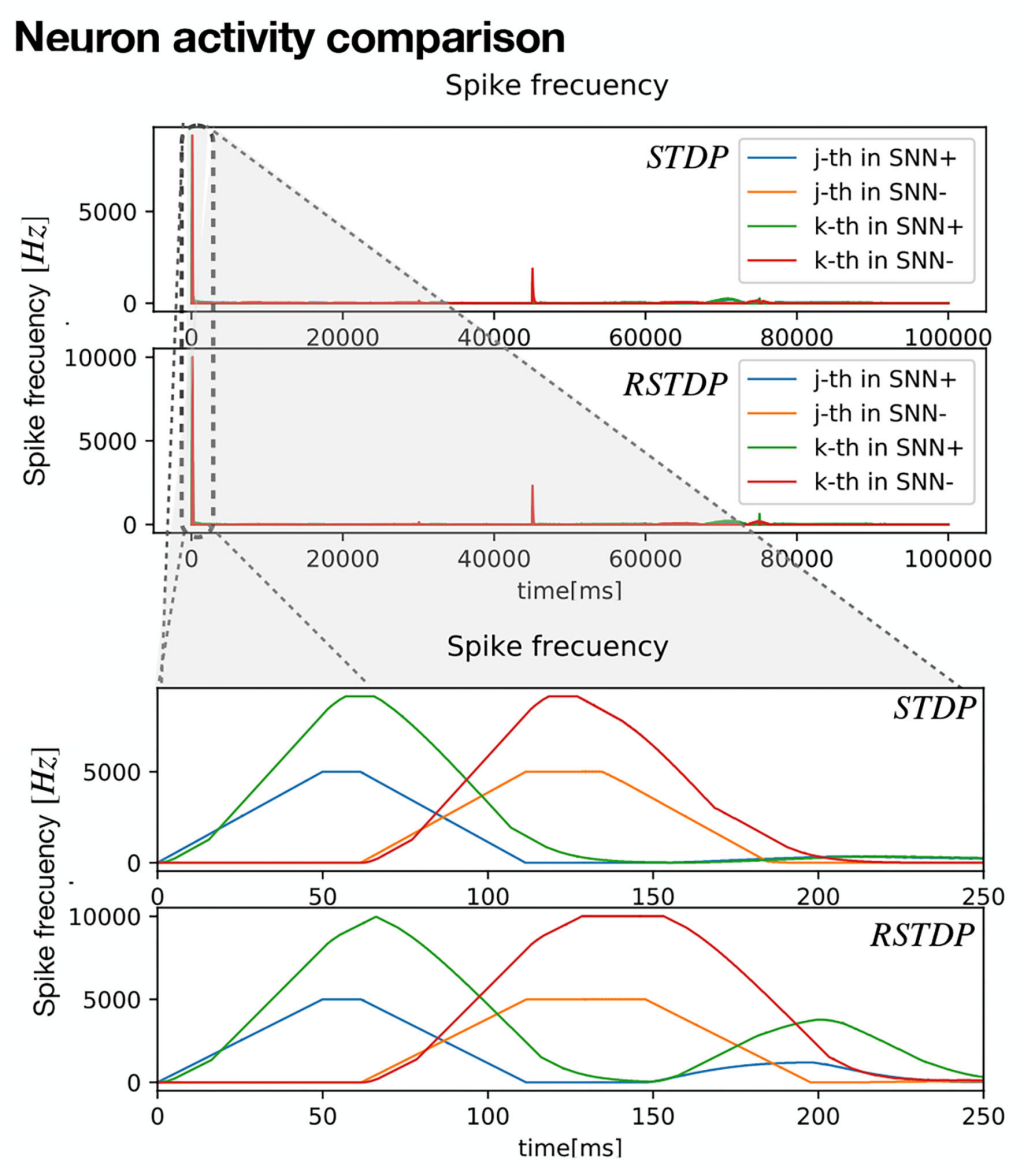
Neuron activity for 1-link controller pendulum. First, *t* = 100*s* of the entire control execution is shown, for SNN-STDP (Up) and SNN-RSTDP (down). A zoom at *t* = 0*ms* − 250*ms* is shown at the highest neuron activity.

Figure 7 shows the spiking frequency of each neuron for both STDP and RTDP controllers. It can be seen that for the first 200*ms* (Refer to zoomed section), the frequency grows and drops quickly, as decoders send current to each input neuron according to the sensibility *s*_*th*_. All the values used for RSTDP synapses, LIF neuron model, and SF encoding and decoding are depicted in Table 1. 1-link Robot simulation parameters are shown in Table 5. The simulation has been performed using Python3 scripts.

**Table 5:** 1-link robot simulation parameters.

**Model**	**Parameters**	**Value**
1-DOF robot	Joint angle boundaries	[θ_*max*_, θ_*min*_] = [0,2π]
parameters	Mass	*M* = 1 *kg*
	New mass at 45*s*	*M*_*new*_ = 3 *kg*
	Longitude	*L* = 2.5 *m*
	Gravity acceleration	*g* = 9.8 *m*/*s*^2^
	Initial viscous friction constant *k*	0.1 *kg*/*s*
	New viscous friction constant at 45*s k*_*new*_	0.5 *kg*/*s*
PID controller	Proportional gain	*k*_*p*_ = 180
	Integral gain	*k*_*i*_ = 50
	Derivative gain	*k*_*d*_ = 12.5
Simulation	Time step	*dt* = 0.1 *ms*
parameters	Number of episodes	6
	Length of an episode	15*s*
	Total time simulation	100*s*
SF encoder	Threshold	*s*_*th*_ = 0.001
	Initial base value	*s*_*b*_ = 0
	Output current	*I*_*c*_ = 140*nA*
SF decoder	Threshold	*s*_*th*_ = 0.2
	Initial base value	*s*_*b*_ = 0

## 4. Discussion

The utility of a neuromorphic controller operation for a 1-DOF robot capable of learning the changing dynamics has been experimentally demonstrated with results comparable with a standard control technique. The PID used for comparison is tuned using a pretty standard and popular procedure for industrial applications. It is an iterative process that intends to eliminate response oscillations based on select proper gains throughout multiple testing executions in the plant. The procedure ends when the responsible technician is pleased with the performance, making it as precise as its interpretation, and it has to be re-tuned each time the dynamics of the plant change.

Unlike the PID, our proposal eliminates the need for tuning procedures. Nonetheless, some issues have to be addressed. First, SNN parameters were selected to mimic biological brain systems, which can be modified to fit actual electrical circuit standards. For example, values of *w*_*min*_ and *w*_*max*_ were chosen arbitrarily, while they should be scaled to fit actual memristor conductance limit values.

*A*_+_, *A*_−_, which control LTD and LTP, play an important role in stability, as they control the learning rate of the system. Small values will result in slow convergence, while larger values will overshoot the output control signal. Value *s*_*th*_ for SF encoding will determine its sensitivity against the input signal, setting the amount of neural activity (spikes) as the response. For decoding, *s*_*th*_ determines output modification, as it has to be sufficiently large to scale the outgoing signal and sufficiently small to avoid overshoot and under-damping behavior. SF encoding also shows no neural activity for the SNN- stage for always increasing signals. *I*_*c*_ modules spiking frequency, as for higher values, output signal amplitude is affected too.

A stability analysis to determine proper LTD, LTP values, thresholds, and current inputs for encoding/decoding and learning rate values is needed. While it is a pending task, some challenges arise, as some system dynamics are not differentiable (LIF, SF models). Therefore, Lyapunov asymptotic stability analysis cannot be performed. However, some possible alternatives are proposing differentiable models of the neuron dynamics, defining the system on the frequency domain, or conducting Von Neumann stability studies.

On the other hand, noise then allows to update synaptic weights constantly, but the sensibility of the encoding is crucial, as for small *s*_*th*_ values, signal variation produces redundant neural activity, generating an accumulative error for decoding. Our proposal effectively used neuron dynamics as a filter, in an open loop. A possible alternative to use or implement alongside would be to use the *Moving-Window SF algorithm* instead. Similar to SF, starting from an initial baseline and threshold values, the baseline is updated differently as an average input signal for a time window. This corresponds to a median filter.

As this scheme proposal tackles fully actuated 1-DOF robotic manipulators, its usage in N-DOF has to be studied. SNN structure might be usable in under-actuated systems, but the SNN architecture must be modified. Hyper-redundant manipulators present a similar problem, as flexible robotic arms can be considered like infinite DOF systems. An infinite neural structure generation is problematic. Therefore, modifications have to be proposed in the future.

## 5. Conclusion

A self-tuning SNN architecture for a 1-DOF manipulator robot arm is proposed, based on a typical control scheme. Numerical simulation shows the feasibility and, in some cases, outperforms PID performance. The architecture also shows self-tuning properties on changing dynamics. From the control theory point of view, a neural structure with similar PID performance is described. Nevertheless, stability analysis is still pending, describing the relationship between spiking activity, current injection, learning rate, and coding velocity. Besides, explainable neural networks are possible, considering control loop architectures. However, neuron models, synapses, and coding/decoding modules should be implemented in analog circuit counterparts to achieve real-time computing scenarios with efficient energy consumption.

## Data Availability Statement

The datasets presented in this study can be found in online repositories. The name of the repository and accession number can be found below: Github, https://github.com/AlejandroJuarezLora/Frontiers-SNN.git.

## Author Contributions

AJ-L proposed, developed, programmed the neural control code and conducted simulation runs, and wrote the first draft of the manuscript. VP-P and HS proposed modifications to the SNN architectures. ER-E reviewed the code required for the PID controller experiments. All authors contributed to the conception and design of the study and manuscript revision, read, and approved the submitted version.

## Funding

The authors would like to thank the economic support of the projects SIP 20210124, 20221780, 20211657, 20220268, 20212044, 20221089, 20210788, 20220226, and COFAA and CONACYT FORDECYT-PRONACES 6005.

## Conflict of Interest

The authors declare that the research was conducted in the absence of any commercial or financial relationships that could be construed as a potential conflict of interest. The handling editor JD declared a shared affiliation with the authors at the time of review.

## Publisher's Note

All claims expressed in this article are solely those of the authors and do not necessarily represent those of their affiliated organizations, or those of the publisher, the editors and the reviewers. Any product that may be evaluated in this article, or claim that may be made by its manufacturer, is not guaranteed or endorsed by the publisher.

## References

[B1] Bing Z. Baumann I. Jiang Z. Huang K. Cai C. Knoll A. (2019a). Supervised learning in snn via reward-modulated spike-timing-dependent plasticity for a target reaching vehicle. Front. Neurorobot. 13, 18. 10.3389/fnbot.2019.00018 31130854 PMC6509616

[B2] Bing Z. Jiang Z. Cheng L. Cai C. Huang K. Knoll A. (2019b). End to end learning of a multi-layered snn based on r-stdp for a target tracking snake-like robot, in 2019 International Conference on Robotics and Automation (ICRA), 9645–9651.

[B3] Chen X. Zhu W. Dai Y. Ren Q. (2020). A bio-inspired spiking neural network for control of a 4-dof robotic arm, in 2020 15th IEEE Conference on Industrial Electronics and Applications (ICIEA) (Kristiansand: IEEE), 616–621.

[B4] Craig J. J. (1986). Introduction to Robotics: Mechanics and Control. Reading, MA: Addison-Wesley Publishing Co. Inc.

[B5] Dupeyroux J. (2021). A toolbox for neuromorphic sensing in robotics. arXiv [Preprint]. Available online at: https://arxiv.org/abs/2103.02751

[B6] Framaux N. Gerstner W. (2016). Neuromodulated spike-timing-dependent plasticity, and theory of three-factor learning rules. Front. Neural Circ. 9, 85. 10.3389/fncir.2015.00085 26834568 PMC4717313

[B7] Gerstner W. Lehmann M. Liakoni V. Corneil D. Brea J. (2018). Eligibility traces and plasticity on behavioral time scales: experimental support of NeoHebbian three-factor learning rules. Front. Neural Circ. 12, 53. 10.3389/fncir.2018.00053 30108488 PMC6079224

[B8] Guo W. Fouda M. E. Eltawil A. M. Salama K. N. (2021). Neural coding in spiking neural networks: a comparative study for robust neuromorphic systems. Front. Neurosci. 15, 638474. 10.3389/fnins.2021.638474 33746705 PMC7970006

[B9] Hao Y. Huang X. Dong M. Xu B. (2020). A biologically plausible supervised learning method for spiking neural networks using the symmetric stdp rule. Neural Netw. 121, 387–395. 10.1016/j.neunet.2019.09.007 31593843

[B10] Hu T. Lin X. Wang X. Du P. (2022). Supervised learning algorithm based on spike optimization mechanism for multilayer spiking neural networks. Int. J. Mach. Learn. Cybern. 10.1007/s13042-021-01500-8

[B11] Izhikevich E. (2004). Which model to use for cortical spiking neurons? IEEE Trans. Neural Netw. 15, 1063–1070. 10.1109/TNN.2004.832719 15484883

[B12] Kasabov N. Scott N. M. Tu E. Marks S. Sengupta N. Capecci E.. (2016). Evolving spatio-temporal data machines based on the neucube neuromorphic framework: design methodology and selected applications. Neural Netw. 78, 1–14. 10.1016/j.neunet.2015.09.011 26576468

[B13] Kendall J. D. Kumar S. (2020). The building blocks of a brain-inspired computer. Appl. Phys. Rev. 7, 011305. 10.1063/1.5129306

[B14] Kheradpisheh S. R. Masquelier T. (2020). Temporal backpropagation for spiking neural networks with one spike per neuron. Int. J. Neural Syst. 30, 2050027. 10.1142/S0129065720500276 32466691

[B15] Kim H. Mahmoodi M. R. Nili H. Strukov D. B. (2021). 4k-memristor analog-grade passive crossbar circuit. Nat. Commun. 12, 5198. 10.1038/s41467-021-25455-0 34465783 PMC8408216

[B16] Lu H. Liu J. Luo Y. Hua Y. Qiu S. Huang Y. (2021). An autonomous learning mobile robot using biological reward modulate stdp. Neurocomputing 458, 308–318. 10.1016/j.neucom.2021.06.027

[B17] Lynch K. (2017). Modern Robotics: Mechanics, Planning, and Control. Cambridge, United Kingdom; New York, NY: Cambridge University Press.

[B18] Mohemmed A. Schliebs S. Matsuda S. And Kasabov N. (2012). Span: spike pattern association neuron for learning spatio-temporal spike patterns. Int. J. Neural Syst. 22, 1250012. 10.1142/S0129065712500128 22830962

[B19] Morrison A. Diesmann M. Gerstner W. (2008). Phenomenological models of synaptic plasticity based on spike timing. Biol. Cybern. 98, 459–478. 10.1007/s00422-008-0233-1 18491160 PMC2799003

[B20] Ogata K. (2010). Modern Control Engineering. Boston, MA: Prentice-Hall.

[B21] Petro B. Kasabov N. Kiss R. M. (2020). Selection and optimization of temporal spike encoding methods for spiking neural networks. IEEE Trans. Neural Netw. Learn. Syst. 31, 358–370. 10.1109/TNNLS.2019.2906158 30990446

[B22] Saxena V. Wu X. Srivastava I. Zhu K. (2018). Towards neuromorphic learning machines using emerging memory devices with brain-like energy efficiency. J. Low Power Electron. Appl. 8, 34. 10.3390/jlpea8040034

[B23] Shi C. Lu J. Wang Y. Li P. Tian M. (2021). Exploiting memristors for neuromorphic reinforcement learning, in 2021 IEEE 3rd International Conference on Artificial Intelligence Circuits and Systems (AICAS) (Washington DC: IEEE), 1–4.

[B24] Spong M. W. Hutchinson S. Vidyasagar M. (2005). Robot Modeling and Control. Wiley. 146–189.

[B25] Taherkhani A. Belatreche A. Li Y. Cosma G. Maguire L. P. McGinnity T. (2020). A review of learning in biologically plausible spiking neural networks. Neural Netw. 122, 253–272. 10.1016/j.neunet.2019.09.036 31726331

[B26] Valadez-Godínez S. Sossa H. Santiago-Montero R. (2020). On the accuracy and computational cost of spiking neuron implementation. Neural Netw. 122, 196–217. 10.1016/j.neunet.2019.09.026 31689679

[B27] Voelker A. R. Eliasmith C. (2020). Programming Neuromorphics Using the Neural Engineering Framework. Singapore: Springer Singapore.

[B28] Yue K. Parker A. C. (2019). Analog neurons with dopamine-modulated stdp, in 2019 IEEE Biomedical Circuits and Systems Conference (BioCAS) (Nara: IEEE), 1–4.

[B29] Zamarreño-Ramos C. Camuñas-Mesa L. A. Pérez-Carrasco J. A. Masquelier T. Serrano-Gotarredona T. Linares-Barranco B. (2011). On spike-timing-dependent-plasticity, memristive devices, and building a self-learning visual cortex. Front. Neurosci. 5, 26. 10.3389/fnins.2011.00026 21442012 PMC3062969

[B30] Zhang X. Lu J. Wang Z. Wang R. Wei J. Shi T.. (2021). Hybrid memristor-cmos neurons for *in-situ* learning in fully hardware memristive spiking neural networks. Sci. Bull. 66, 1624–1633. 10.1016/j.scib.2021.04.014 36654296

